# Hepatic Encephalopathy and Melatonin

**DOI:** 10.3390/antiox11050837

**Published:** 2022-04-25

**Authors:** Archana Arjunan, Dhiraj Kumar Sah, Young Do Jung, Juhyun Song

**Affiliations:** 1 Department of Anatomy, Chonnam National University Medical School, Hwasun 58128, Korea; archanaibms@gmail.com; 2 Department of Biochemistry, Chonnam National University Medical School, Hwasun 58128, Korea; 197784@chonnam.edu; 3 BioMedical Sciences Graduate Program (BMSGP), Chonnam National University, 264 Seoyangro, Hwasun 58128, Korea

**Keywords:** melatonin, hepatic encephalopathy, hyperammonemia, neurotransmitter, neuroinflammation, cognitive impairment

## Abstract

Hepatic encephalopathy (HE) is a severe metabolic syndrome linked with acute/chronic hepatic disorders. HE is also a pernicious neuropsychiatric complication associated with cognitive decline, coma, and death. Limited therapies are available to treat HE, which is formidable to oversee in the clinic. Thus, determining a novel therapeutic approach is essential. The pathogenesis of HE has not been well established. According to various scientific reports, neuropathological symptoms arise due to excessive accumulation of ammonia, which is transported to the brain via the blood–brain barrier (BBB), triggering oxidative stress and inflammation, and disturbing neuronal-glial functions. The treatment of HE involves eliminating hyperammonemia by enhancing the ammonia scavenging mechanism in systemic blood circulation. Melatonin is the sole endogenous hormone linked with HE. Melatonin as a neurohormone is a potent antioxidant that is primarily synthesized and released by the brain’s pineal gland. Several HE and liver cirrhosis clinical studies have demonstrated impaired synthesis, secretion of melatonin, and circadian patterns. Melatonin can cross the BBB and is involved in various neuroprotective actions on the HE brain. Hence, we aim to elucidate how HE impairs brain functions, and elucidate the precise molecular mechanism of melatonin that reverses the HE effects on the central nervous system.

## 1. Introduction

The liver, which is a metabolic organ, is involved in detoxification, nutritional metabolism, maintenance of blood volume, and hormone regulation [1]. Hepatic disease and liver failure are the leading cause of death worldwide [2], and are involved in the development and pathogenesis of neurological illnesses [3]. Globally, 40% of liver cirrhosis cases transition to hepatic encephalopathy (HE) (also known as portosystemic encephalopathy (PSE)). HE is a severe metabolic disorder caused by end-stage liver disease [4] and associated with reversible neurological dysfunction ranging from personality changes to coma and death [5]. HE is categorized into two classes: 1. Covert HE/Minimal Hepatic encephalopathy (MHE), which is associated with neuropsychiatric symptoms, including alteration in mood, personality, memory, sleep, and motor coordination; and 2. Overt HE, which occurs when covert HE becomes chronic, causing a decline in the patient’s survival [6]. HE can also be classified into three types according to the causes: Type I, acute liver failure (ALF)-induced HE; Type II, Bypass shunts-induced HE; and Type III, Chronic liver disease-induced HE. Notably, HE does not have a single clinical symptom. HE may either be accompanied with reversible metabolic encephalopathy, atrophy, or edema in the brain [7].

**Figure 1:**
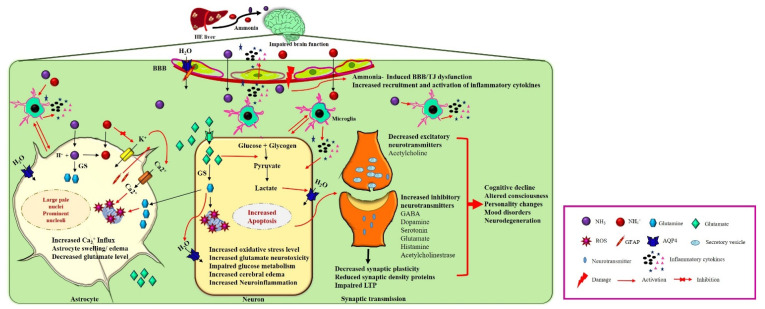
**Neuropathogenesis of HE on brain dysfunction**. HE liver releases excess nitrogenous toxin (NH_3_, NH_4_^+^) that enters cerebral circulation. Ammonia can cross the BBB and trigger the other pathological response such as activation of aquaporin water channels and damage of BBB’s tight junctions. Astrocytes detoxify ammonia to form glutamine from glutamate by glutamine synthase (GS). Excess glutamine production increases oxidative stress, aquaporin channels’ activation, increases Ca^2+^ influx and GFAP production, and decreases glutamate uptake leading to accumulation of glutamate into the extracellular fluid. Activation of water channels, increased Ca^2+^ influx, and increased glutamine secretion cause astrocyte swelling. On the other hand, accumulated extracellular glutamate enters into neurons, causing glutamate neurotoxicity. Intracellular glutamate impairs glucose metabolism, activates microglial inflammatory cytokines, increases oxidative stress, and inhibits mitochondrial functions, leading to decrease in excitatory neurotransmitters synthesis and release into the synapses. In synaptic transmission increases synthesis and release of inhibitory neurotransmitters impairing the LTP, synaptic plasticity, and reducing synaptic density proteins, leading to cognitive decline and other neuropsychiatric illnesses.

The pathophysiology of HE is multifactorial and has not been clearly explained. Various in vivo and in vitro liver failure studies demonstrated that large amounts of ammonia crosses the blood–brain barrier (BBB), causing neuropathological disruptions, such as personality changes, altered cognition, locomotor ability, and consciousness [8]. Ammonia is the central metabolite and the principal neurotoxin related to HE [9]. Ammonia is synthesized in enterocytes from glutamine and metabolized by the liver [9]. According to previous studies, normal healthy individuals have 45 μM ammonia in arterial circulation [10]. The highest ammonia concentration is found in end-stage liver disease with irreversible brain damage (340 μM) [11]. In liver diseases, liver detoxification unexpectedly declines, causing hyperammonemia [9]. The circulatory ammonia enters into the brain and deposits in the brain and cerebrospinal fluid (CSF) [12]. The accumulated neurotoxin increases oxidative stress (OS) [13], generating proinflammatory cytokines [14], altering the synthesis and transmission of the neurotransmitters [15], impairing glucose and energy metabolism [16], and inducing astrocyte swelling [17] and brain edema [18] (Figure 1).

A therapeutic approach for HE is currently emerging as an important issue. Thus, therapies that inhibit oxidative stress induced by hyperammonemia are markedly needed to inhibit neuronal damage caused by oxidative stress and enhance the prognosis of HE. Various HE clinical and experimental reports revealed several pharmacologic therapies, such as antibiotics [19] and nutritional supplements [20] for HE. However, other studies revealed the adverse effects of antibiotics in HE [21]. Currently, overt HE can only be treated, while covert/MHE does not have an appropriate therapeutic approach in modern medicine. Therefore, modern medicine focuses on the HE therapeutic agent that recovers the hepatic/neuronal functions with minimal adverse effects. Mainly, neurosteroids/endogenous hormones may demonstrate a neuroprotective action on the nervous system. These biological compounds diminish oxidative stress, inflammation, excitotoxicity, brain edema, and neurodegeneration [22]. Few endogenous hormones (estrogen, progesterone, and lipoic acid) are involved in neuroprotection [23]. Precisely, melatonin was found to exhibit a promising neuroprotective effect on HE [1]. Several patients with cirrhosis and HE have severely impaired melatonin metabolism [24], with altered melatonin secretion and circadian patterns. Melatonin is the sole endogenous hormone linked with HE. However, interpreting their pathophysiology remains finite, and their therapy is challenging. Here, we aim to recapitulate the multiple effects of melatonin in the HE brain.

## 2. Melatonin in the CNS

**Figure 2:**
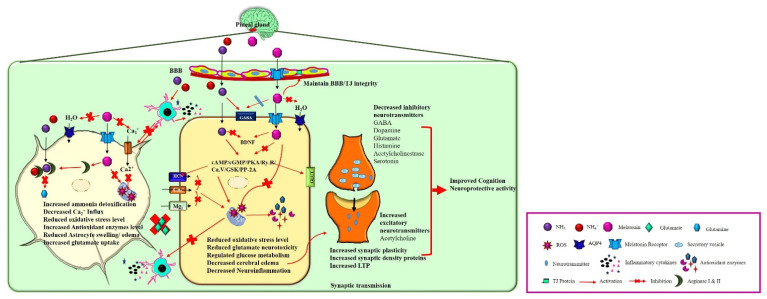
**Neuroprotective action of melatonin on HE brain**. In the brain, melatonin is synthesized and released from pinealocytes of the pineal gland. Melatonin binds to its receptors and activates various physiological functions such as 1. In astrocytes: Melatonin detoxifies the excess ammonia by activating the Arginase I and II enzyme that prevents glutamine synthesis and glutamate accumulation in extracellular fluid. Furthermore, melatonin prevents neuroinflammation and astrocyte swelling by decreasing the Ca^2+^ influx and inhibiting water channel activation. 2. In a neuron, melatonin inhibits the cAMP/cGMP/PKA/Ry.R/Ca.V/GSK/PP-2A signaling pathway leading to the decreased oxidative stress level, inhibits the microglial activation, and reduces the inhibitory neurotransmitter synthesis and release. Moreover, melatonin regulates glucose metabolism by acting on insulin/GLUT receptors, facilitating synaptic plasticity, LTP, cognition by increasing the synaptic density proteins expression, and increasing the excitatory neurotransmitter release.

Melatonin (N-Acetyl-5-methoxytryptamine), a neurohormone, is known as an “internal synchronizer” involved in circadian rhythms [25]. Melatonin is synthesized in the pinealocytes and its derivatives are produced by the retina, astrocytes, kidney, lymphocytes, platelets, and skin [26]. Tryptophan is the vital precursor during melatonin synthesis, which is dependent on the light and dark cycle [27]. Hydroxylation and decarboxylation of tryptophan results in serotonin, and the acetylation of serotonin forms N-acetyl serotonin (NAS) by N-acetyltransferase [27]. Hydroxyindole-O-methyltransferase (HIOMT)/acetylserotonin methyltransferase then converts NAS to melatonin [27]. Melatonin synthesis merely relies on the precursors, enzyme availability, and seasonal and circadian rhythms [27]. Melatonin is a chronobiotic molecule that is not merely confined to circulation and augments to enable direct impacts in the central nervous system (CNS) [28]. Melatonin also acts as a circadian pacemaker, and this pleiotropic controller has numerous physiological roles, including in the sleep–wake cycle, neuro-immuno endocrine, and circadian rhythm [29,30]. Melatonin initiates the signaling pathway by binding to melatonin receptors (MT (1,2,3)) [31]. Both G-coupled transmembrane MT1 and MT2 receptors are predominantly located in the brain and other extra pineal tissues (liver, bone, and retina). MT3 is identified in the liver, kidneys, heart, adipose tissue, and brain. The activated MT receptors trigger various signaling and transcriptional pathways and act as a neuroprotective agent in various CNS disorders. These receptors are also involved in the pathology and chief drug target for CNS disorders. Melatonin can cross the BBB and protect against brain injury (neurodegenerative diseases, trauma, hypoxia, and HE) [32,33] by acting as a potent anti-inflammatory [34,35], anti-apoptotic [36], antioxidative [37,38], anti-tumor [30], anti-diabetic, anti-obese, neuroprotective, cardioprotective, and mood-stabilizing agent [28]. Collectively, melatonin has various potentials for treating both systemic pathology and neuropathology based on their characters (Figure 2).

## 3. Hepatic Encephalopathy (HE) and Melatonin (Hyperammonemia)

The neuropathogenesis of HE remains unclear. The complications of HE include glutamine, chronic infections, and profuse gastrointestinal bleeding, and causes elevated ammonia levels in the blood and CNS [9]. According to the prevailing hypothesis of HE, gut-derived nitrogenous toxins of ammonia can cross the BBB and induce neurological symptoms [2]. The biochemical analysis in numerous clinical and experimental studies has confirmed increased circulatory ammonia levels in HE [13]. HE experimental models can also be created by increasing the ammonia level in blood circulation [39,40].

In this review, ammonia is defined as the concentration of both ammonia (NH_3_) and ammonium ion (NH_4_^+^). NH_3_ is a lipophilic compound that can cross the plasma membrane, while NH_4_^+^ is transported through ionic channels [41]. Ammonia is derived from all amino acids, nucleic acids, and renal glutamine. Ammonia is also produced by normal floral bacterial enzymes within the gastrointestinal tract (3–4 mg/day) [41] and is metabolized by bacterial enzymes in the gastrointestinal tract and in the liver via the urea cycle. A high level of ammonia crosses the BBB, which leads to oxidative stress, alters glucose and neurotransmitter metabolism, and disrupts of neuronal functions and structure, such as astrocyte swelling in HE [42].

Based on different studies, melatonin is a potent hepato-neuroprotector against hyperammonemia (Table 1). The liver is the principle organ involved in nitrogen homeostasis. Hepatic disease leads to impaired urea cycle, ammonia trafficking, and hyperammonemia [43]. In the urea cycle, ammonia is detoxified by five enzymes (arginase, argininosuccinate synthetase, argininosuccinate lyase, carbamyl phosphate synthetase I (CPS-I), and ornithine carbamyl transferase) [44]. Arginase is the final process enzyme that converts L-arginine to l-ornithine/urea to degrade the nitrogenous toxin of ammonia [45]. There are two types of arginase in mammals: 1. cytosolic arginase I, which is expressed in the liver (>98%); and 2. mitochondrial arginase II, which located in extrahepatic tissues (2%) (renal, brain, lung, intestine, and breast) [45]. According to Aydogdu et al., melatonin enhances arginase (I and II) expression and reduces the level of nitric oxide (NO) [46]. Another study revealed that melatonin reduces the metabolite accumulation end products, such as ornithine (Orn), homocitrulline (Hcit), and ammonia, in the urea cycle, owing to its antioxidant defense in hyperornithinemia–hyperammonemia–homocitrullinuria syndrome (HHH) [47]. Studies demonstrated that a high ammonia (>500 μM) level generates the free radical production in the cellular level [48]. However, hyperammonemia was found to alter mitochondrial functions by increasing the free radical production (LPO)/reactive oxygen species (ROS), decreasing adenosine triphosphate (ATP) synthesis [49] and disturbing cellular pH by reducing a-ketoglutarate [50].

Numerous in vivo and in vitro studies confirmed the antioxidant activity of melatonin on oxidative stress-induced damage [54]. In HE, the melatonin demonstrated antioxidative properties by inhibiting ammonia-induced free radical production [1,55,56]. In various oxidative stress markers, 3-nitrotyrosine is the main oxidative stress diagnostic marker (90% sensitivity and specificity) for MHE [57]. Hence, various scientific reports mentioned that melatonin inhibits the 3-nitrotyrosine generation induced by oxidative stress models [58,59]. Moreover, melatonin inhibits NO production by converting into NAS to reduce the oxidative stress [60]. As a result, these functions of melatonin on ammonia metabolism-related enzyme arginase and metabolites leads to the reduction of ammonia accumulation. Melatonin reduces the oxidative stress induced by hyperammonemia by generating antioxidants, and instantly scavenging ROS.

## 4. HE and Melatonin (Neuroinflammation and BBB Disruption)

HE is known to affect astrocyte dysfunction by making hyperammonemia toxicity [61]. In this review, we explain the paradigm of neuroglial communication, which is reconstructed by melatonin in HE. Several studies have affirmed that the accumulation of toxic metabolites alters cell signaling by facilitating the activation of microglia, neuroinflammation, and Alzheimer Type II astrocytosis, and plays an important key role in HE [61,62].

In the CNS, astrocytes impact the formation and maintenance of the BBB [63], and regulate cerebral blood flow, water channel expression [64], neurotransmitter release, and reuptake [65]. Microglia are immune cells that act as housekeeping factors and modulators of neuroinflammation [66]. Under physiological conditions, microglia monitor myelin homeostasis [67], synaptic activity, pathogen entry, and injury. However, under pathological conditions, microglia triggers neuroinflammation by increasing cytokines and chemokines [68]. Ammonia has multiple toxic impacts on cellular metabolisms, such as the production of free radicals by tricarboxylic acid (TCA) cycle enzymes, malate-aspartate shuttle, mitochondrial respiratory chain inhibition, and increase in glutamine to induce cell swelling [69]. These toxic attributes will be discussed later.

Astrocytes is a vital element of the BBB and regulates the arachidonic acid-dependent pathway to maintain cerebral blood flow (CBF) [70]. Astrocytes can also uptake and metabolize 7% of arterial ammonia [10]. Ammonia (NH_3_) crosses the BBB via passive diffusion to astrocytes [71]. In HE, the levels of blood ammonia, cytokines, transforming growth factor-beta (TGFβ1), tumor necrosis factor (TNF), matrix metalloproteinase 9 (MMP-9), and bile acids are increased [72]. Elevated MMP-9, TNF level, and bile acids impair the BBB’s tight junction (TJ) proteins, such as occludin and claudin-5 [72]. Damaged TJ allows the influx of ammonia. The accumulation of ammonia and bilirubin also reduces the BBB’s breast cancer resistance protein (BCRP) expression, which protects the brain from the toxin [72]. Astrocytes catalyze the glutamine formed from ammonia, which is converted to glutamate and NH_4_^+^ by glutamine synthetase [73]. In contrast, glutaminase converts glutamine to glutamate and stores it as a neurotransmitter in neurons for reuptake by astrocytes [71,74]. The osmolyte property of glutamine increases oxidative stress by activating mitochondrial pore transition in the mitochondria [75], and these factors are the main reason for astrocyte swelling and cerebral edema. Hyperammonemia over-activates the Na-K-2Cl cotransporter (NCCa-ATP) channel [76], increases ionic influx into the astrocyte, alters the water concentration gradient, and activates aquaporin 4 (AQP4) water channels [77], causing astrocyte swelling and brain edema. On the contrary, ammonia activates tryptophan metabolites, and induces ROS production, Ca^2+^ influx, NADPH oxidase, and mitochondrial pore transition [78] caused by oxidative stress. Finally, these mechanisms increase ROS and astrocyte senescence. ROS generation initiates p53 phosphorylation at serine 392 through mitogen-activated protein kinases (p38MAPK) [17,61]. Several studies confirmed that hyperammonemia activates the secretion of inflammatory cytokines (interleukin-6 (IL-6), interleukin-1beta (IL-1β), interferon gamma (IFN-γ), and tumor necrosis factor α (TNFα) in ammonia-induced astrocyte cultures [50]. These cytokines further activate nuclear factor kappa-light-chain-enhancer of activated B cells (NF-κB) [79]. Similar to increased nitric oxide synthase (iNOS), IL-1β and hemoxygenase-1 (HO-1) were found to increase in ammonia-induced astrocyte cultures [80]. These findings indicate a direct connection between inflammatory cytokines, ROS, and ammonia in HE associated with astrocyte swelling and cerebral edema [61]. Thus, studies proved that astrocytes are crucial glial cells that link ammonia and inflammation by unlocking the BBB via an arachidonic acid-dependent mechanism [81]. Astrocytes demonstrated the pattern of Alzheimer’s Type II astrocytosis to have prominent nucleoli and large pale nuclei characters, whichare found in white and gray matter in the HE brain [62].

In astrocytes, lactate dehydrogenase (LDH)-1 and LDH-5 expression levels are markedly enhanced due to hyperammonemia [82]. Further, reduced glucose utilization causes ATP depletion and TCA cycle enzyme (α-ketoglutarate dehydrogenase) inhibition [9]. Therefore, excess deposition of lactate induces cytotoxic edema known as astrocyte swelling. A recent study hypothesized that the swelling of astrocytes is caused by glial fibrillary acidic protein (GFAP) in HE [65]. Langer et al. reported that the protein expression of GFAP is reduced in the ALF rat cortex [83]. GFAP reduction alters the visco-elastic nature of astrocytes, causing astrocyte swelling and brain edema. Other studies also reported that astrocyte swelling and brain edema are caused by the reduction of protein and gene expression of a water channel (aquaporin II), glucose transporter 1 (GLUT-1) [84], and GFAP [85] in HE.

Microglial activation is the second key factor for neuroinflammation in HE. In AHE/chronic hepatic disease, the increased level of ammonia, TCA, TGFβ1, and TNF interacts with neuronal receptors and increases C-C Motif Chemokine Ligand 2 (CCL2) production, which is followed by microglial activation [1]. Activated microglia can release proinflammatory cytokines (TNFα, IL-1α, IL-1β, and IL-6), other inflammatory markers (Toll-like receptor 4 (TLR4), OX-42, OX-46, CD11b), and numerous inflammatory signaling pathway factors (NF-kB, mitogen-activated protein kinase (MAPK) p53, and NO/cGMP pathway) [40], which are involved in neuropathogenesis-induced HE [61,86]. Alternatively, neuroinflammation can also be triggered by hyperammonia-induced oxidative stress within astrocytes and neurons [50]. Oxidative stress and cerebral edema alter the physiological functions of astrocytes [87] and inhibit neuronal-glial cell communication, leading to symptoms of HE [88].

Melatonin is a potent immunomodulator with diverse functions. Melatonin has a conceivable function in inhibiting the activation of the pro-inflammatory cytokines in the MAPK and NF-κB pathways [89]. An injection of melatonin reduces BBB permeability and brain edema in an in vivo and in vitro model [90]. Based on the cell requirement, melatonin acts as anti/pro-inflammatory agent and regulates immunological responses [91]. Melatonin exerts its anti-inflammatory activity by blocking iNOS, cyclooxygenase -2 (COX-2), and NOD-, LRR-, and pyrin domain-containing protein 3 (NLRP3) expression [92,93]. According to Permpoonputtana et al., melatonin inhibits TNFα mRNA expression, phosphorylated p65 NF-κB, and nuclear factor erythroid-2-related factor 2 (Nrf2) in dopamine SH-SY5Y cell lines [94]. Melatonin also maintains the BBB integrity mediated via the TLR4/NF-κB-signaling pathway [95]. To sum up, melatonin acts as potent anti-inflammatory agent and maintains the BBB integrity by inhibiting the neuroinflammatory pathways (TLR4/NF-κB, MAPK pathways) and microglial activation, as well as maintaining the tight junction proteins’ integrity and inhibiting the astrocyte swelling brain edema by inactivating the ammonia-induced AQP4 channels.

## 5. HE and Melatonin (Neurotransmitters)

As there is evidence of glial activation and neuroinflammation in HE, neurotransmitters should be reviewed to understand the pathologies of HE. The major excitatory neurotransmitter related to HE is glutamate [13]. Previously, we explained the synthesis and metabolism of glutamate in astrocytes. Experimental HE studies revealed that glutamate’s release is increased in extracellular fluid and leads to hyperammonemia [96]. Ammonia directly influences glutamatergic neurotransmission [97]. Further, studies have suggested that hyperammonemia boosted the secretion of glutamate from astrocytes [98]. Astrocyte swelling has an impact on the release of glutamate by regulating a pH and Ca^2+^-dependent mechanism [99]. Ammonia affects the expression of N-methyl-D-aspartate (NMDA) receptors, and controls α-amino-3-hydroxy-5-methyl-4-isoxazolepropionic acid (AMPA) receptor-mediated currents [100]. Ammonia also decreases the depolarization caused by NMDA and AMPA receptors and reduces the production of inositol-3-phosphate (IP_3_) [101]. These findings indicate that ammonia has a direct impact on glutamatergic transmission in the neuronal cell synapse. The neuroprotective effect of melatonin against glutamate neurotoxicity has been demonstrated in various clinical and experimental studies via reduced NO production, decreased Ca^2+^ influx [102], and antioxidative signaling [103,104].

The level of gamma-aminobutyric acid (GABA) as an inhibitory neurotransmitter is increased in HE [100]. In fact, the increased GABAergic tone is the principal neuropathology of HE [100]. In HE, hyperammonemia increases GABA release and activates the peripheral benzodiazepine (PTBR) receptors [105]. PTBR and diazepam binding inhibitor (DBI) are increased in astrocytes and CSF [106]. Furthermore, the clinical report of HE comatose patients revealed that the upregulation of PTBR receptors increased astrocyte swelling [107,108] (II astrocytosis) [109]. Activated PTBR initiates the de novo synthesis of the neurosteroid/neuroinhibitor 3a-5a-tetrahydro-progesterone (Allopregnanolone) [110]. In the brain of the deceased HE coma patients’ brain, increased allopregnanolone was found, which increased the GABA-recruited chloride currents [62]. In contrast, acetylcholine inhibits the GABA receptor-mediated inhibitory effects. In HE, acetylcholinesterase levels are high, thereby catalyzing acetylcholine in the synaptic cleft [111,112]. To confirm these findings, one study has demonstrated that the administration of acetylcholine reverses is beneficial on the coma in HE patients [113]. The antagonist of these neurosteroids is the potential target for HE [22]. Numerous studies reported that melatonin inhibits the expression of GABA and acetylcholinestrase [114,115]. Claudia et al. revealed that melatonin receptors modulate the GABAergic system by inhibiting increased calcium accumulation, which activates GABA in xenopus tectal cells [116]. Cheng et al. reported that melatonin modulates the rat hippocampal GABAergic responses via benzodiazepine (BZ) receptors [117]. Huang et al. found that melatonin inhibited lateral hypothalamic GABAergic neurons via the inhibition of HCN ion channels [118]. Fernandez-Bachiller et al. mentioned that the melatonin hybrid interacts with the peripheral anionic site (PAS) of acetylcholinesterase, which modulates the acetylcholinesterase activity on acetylcholine [119].

Next, another vital neuromodulator in neuropathology is adenosine. Adenosine inhibits the release of postsynaptic neurotransmitters (glutamate, GABA, serotonin, and dopamine), and modulates neuronal excitability [120]. Studies confirmed that the adenosinergic mechanism is disturbed in HE compared to the normal brain [15]. Adenosine receptors (A1, A2_A_, A3) are downregulated in severe HE [15,100]. A1 receptor downregulation causes increased level of glutamate, leading to glutamate neurotoxicity [121]. The downregulation of A2 receptors increases GABA release, which is known as an increased GABAergic tone [122].

Furthermore, some reports have been published on the roles of monoamines in the HE brain [100]. The main monoamine involved in HE neuropathology is a serotonin. A disturbed serotonergic system is observed in many clinical HE conditions [123,124]. Research reports have revealed that melatonin increases serotonin synthesis, and increased serotonin facilitates for melatonin production [125,126]. In contrast, Agrawal et al. reported that melatonin inhibits the serotonin transporter function in the epithelial cells of the intestine. Thus, the melatonin and serotonin interconnection might be confusing the action on melatonin on the HE brain [127].

According previous studies, the levels of cerebral dopamine and its metabolite, homovanillinic acid, increases the HE brain [128,129,130,131]. Melatonin is used as an antidopaminergic agent in Parkinson’s disease (PD) [132]. Dopamine release is inhibited by melatonin, and is demonstrated in diverse brain regions such as the hippocampus, hypothalamus, pons-medulla, and retina [133]. Zisapal et al. mentioned that the dopaminergic pathway is modulated by melatonin and subsequently affects antioxidant responses and mitochondrial activity in PD patients [132]. Striatal BTZ receptors play a major role in controlling dopamine-related neuropathology [134]. A study suggests that melatonin alleviates PD symptoms such as dyskinesia by allosterically interacting with BZ and GABA_A_ receptors [135]. Moreover, melatonin inhibits the cAMP production and significantly regulates the neuropathology induced by the D1 or D2 dopamine receptor agonists [136].

Melatonin inhibits the responses of postsynaptic NMDA-receptors to glutamate that modulates long-term potentiation (LTP) [132,133]. Based on another theory, melatonin receptors mediate dopamine/cAMP signaling, which modulates dopaminergic neurotransmission [137].

In addition, the activation of histamine and its precursors increased in HE [124,138]. These are the main causative factors for depression and sleep disturbances [100]. The link between histamine and melatonin regulates hormonal, neuronal, and behavioral activities [100].

Investigators have proposed the H1HR/CaV1.3/RyR and H1HR/Gβγ/cAMP/PKA/CFTR pathways, which mediate histamine and melatonin [139]. Silva et al. also confirmed that histamine-induced NO generation in endothelial cells was inhibited by melatonin [140]. From the above mentioned, melatonin regulates the neurotransmitter synthesis and secretion by acting on ionic channels (HCN, Ca^2+^, Mg^2+^, and GLUT), acting on receptor (PAS/NMDA/GABA/glutamate), and modulating the signaling pathways (cAMP/cGMP/PKA/Ry.R/GSK/PP-2A).

## 6. HE and Melatonin (Insulin Resistance)

In the CNS, the brain is the insulin-sensitive organ [141] that contains remarkable amounts of insulin binding receptors, especially in cerebral cortex, hypothalamus, and hippocampal post synaptic densities [142,143]. Insulin and glucose uptake regulate various neurophysiological functions, including neurogenesis, synaptic plasticity, and cognition [144]. Impaired insulin and glucose regulation lead to cognitive decline and the development of neurodegenerative diseases [145,146,147]. The liver, which is a metabolic organ, is involved in glucose metabolism (gluconeogenesis, glycogenesis, and glycolysis) [3]. Therefore, altered liver functions or liver diseases impair the glucose metabolism, and might cause glucose metabolic diseases such as diabetes mellitus. Diabetes and insulin resistance are interrelated with HE. In fact, studies have demonstrated that 96% of cirrhotic patients displayed glucose intolerance and 30% had type 2 diabetes (T2DM) [148].

Inconsistent scientific reports suggest that hyperammonemia impairs blood glucose and insulin secretion [149,150,151]. Elevated ammonia increased intermediate metabolites, such as nonesterified fatty acids, glucose, pyruvate, and a-ketoglutarate, and decreased glucose phosphate [152]. A generally known theory is that augmented glutamate dampens the TCA cycle of a-ketoglutarate to interrupt ATP and energy metabolism [153].

According to numerous reports, melatonin regulates insulin secretion by acting on carbohydrate and glucose metabolism [154,155]. Melatonin is also used as an anti-hyperglycemic agent for T2DM [154] and can maintain insulin secretion by acting on three signaling pathways in pancreatic beta cells: 1. MT1 receptor-mediated cAMP/PKA/Ca^2+^ pathway [155], 2. MT2 receptor-mediated cGMP/PKG/Ca^2+^ pathway [156], and 3. MT2 receptor-mediated PLC/IP3/ER/SR/Ca^2+^ or PLC/DAG/PKC/Ca^2+^ pathway [157,158]. Insulin binds to an extracellular insulin receptor (InsR), leading to intracellular β subunit autophosphorylation followed by the activation and phosphorylation of InsR substrate (IRS-1) elements [159]. Liu et al. revealed that 10 mg/kg/day of melatonin from the day of embryonic administration reduced the neural tube defects in embryos (e.g., exencephaly) by activating neural stem cell proliferation and inhibiting apoptosis regulated via the extracellular signal-regulated kinase (ERK) pathway [160]. Furthermore, a various genome-wide association study (GWAS) demonstrated that melatonin’s single nucleotide polymorphism (MTNR1B) is associated with hyperglycemia and T2DM [161,162,163].

These findings strongly suggest that melatonin regulates insulin and glucose metabolism, and may serve as the reason for reduced hyperglycemia-induced hyperammonemia in HE by modulating signaling pathways (cAMP/PKA/Ca^2+^/cGMP/PKC/Ca^2+^ pathway/PLC/DAG/PKC/Ca^2+^ pathway/ERK), and regulating glucose metabolism by acting on its respective receptors (GLUT/InsR).

Given these consequences, melatonin could improve insulin sensitivity and brain function in HE.

## 7. HE and Melatonin (Cognitive Function)

Several studies revealed that the main neuropathological symptoms of HE is a cognitive decline [4,61]. Melatonin has been found to promote cognition in both clinical and experimental studies (Table 2). Astrocyte senescence is intensely linked with oxidative stress and cognitive decline and is observed in HE [61]. Many studies revealed that ammonia inhibits astrocyte growth via arrest in the S-phase of the cell cycle [17]. Ammonia mainly upregulates SA-β-Gal, which is the diagnostic marker for senescence [164].

The mechanisms of astrocyte senescence have not been clearly elucidated; however, based on the main hypothesis, astrocyte senescence decreases synaptic connections [17]. Ammonia-induced astrocyte cultures demonstrated reduced synaptic connections, and are linked with a decreased level of the brain-derived neurotrophic factor (BDNF) and thrombospondins (TSP) [168]. Structural and functional alterations in astrocyte synapses are primarily due to BDNF-induced TrkBT-dependent (Tyrosine Receptor Kinase B) signaling [169]. However, reduced BDNF-actin polymerization induction was found in ammonia-induced astrocyte cultures [17]. Ephrins (Eph)/Ephrin-Receptors (EphR) and BDNF- TrkBT signaling interaction with astrocyte tripartite synapses and neurons intensify the synaptic contacts [170]. Another study revealed the inhibition of Eph/EphR signaling in ammonia-induced astrocyte cultures from the HE patient’s brain [170]. Hence, hyperammonia-induced astrocyte senescence is linked with disturbing synaptic stability/connectivity via the BDNF inhibition, blocking TrkBT-dependent and ephrin/ephrin receptor signaling in the brain [97]. Thus, defective astrocyte senescence and neuronal/glial transmission can lead to persistent morphological alterations in the HE brain, which may proceed for the resolution of overt HE [17].

To support these findings, Gorg et al. reported that astrocyte senescence in an in vitro HE model activated by hyperammonia-induced glutamine synthesis-dependent *O*-GlcNAcylation in an in vitro study [171]. Moreover, ammonia-induced oxidative stress activates the astrocyte senescence by triggering the p53 dependent transcription inhibitory genes (p21, GADD45α) [168]. Therefore, astrocyte senescence is an important key factor that activates neuroinflammation, aging of neuro-glial cells, and causes cognitive decline [172].

Synaptic connection is another hypothesis related to learning and memory [173]. Synapses are a specialized intercellular (functional) approximation between neurons, and synaptic plasticity denotes learning and memory [174]. Synaptic function and synaptic signal transduction are regulated by postsynaptic density (PSD) [173]. In various PSD types, PSD-95 is a vital protein that regulates and integrates synaptic signals, and is linked with cerebral diseases [175]. PSD-95 mediates the learning and memory process by aggregating the N-methyl-D-aspartate receptor (NMDAR) to generate LTP [176,177]. PSD-95 can also transmit neurotoxic signals via NMDAR overexpression [178]. 

Fawad et al. demonstrated the cognitive enhancement activity of melatonin administration (5 mg/kg) in middle cerebral artery occlusion (MCAO) rat models. In this study, melatonin facilitates the NR2a/PSD-95 complex association/PI3K/Akt/GSK3β pathway. Moreover, melatonin boosts the neuroprotective factor of γ-enolase expression and conserves the synaptophysin and SNAP25 presynaptic protein expression and p-GluR1845 postsynaptic protein expression [179]. Furthermore, in HE, the accumulation of ROS, increased glutamate, altered synaptic contacts/morphology, and the LTP leads to cognitive decline [180].

Numerous clinical/experimental studies and meta-analyses demonstrated the neurocognitive effect of melatonin in cognitive decline models [181,182,183]. Melatonin exerts neuroprotection against the cholinergic/serotonergic system and promotes GABAergic neurotransmission [184]. Guermonprez et al. reported that melatonin facilitates the choline and choline acetyltransferase functions of synaptosome/synaptic vesicles [185]. Melatonin administration was found to inhibit GSK-3/PKA/PP-2A activation in the rodent brain [186,187,188]. By using melatonin-treated glutamate-exposed neuronal cultures, Wei et al. demonstrated that MMP-9, PSD-95, and growth-associated protein 43 (GAP-43) proteins were not only upregulated, but facilitated neuronal plasticity in the rodent stroke model [189]. Melatonin was also found to increase BDNF expression via the PLC pathway [190]. 

These studies suggest that melatonin plays an important neurocognitive role in addressing HE-induced cognitive decline. Melatonin is a potent neurocognitive agent by increasing synaptic connectivity, synaptic density proteins, and increasing LTP, inhibiting GSK3β/PKA/PP-2A signaling pathways, decreasing inhibitory neurotransmitter synthesis and release. Melatonin’s neurocognitive effect was confirmed to be due to its antioxidant, anti-inflammatory, and anti-apoptotic properties on HE.

In summary, this review mainly focused on how melatonin communicates with the HE brain (Figure 2). A hallmark of HE is loss of neuro-glial function, which in turn leads to cognitive decline. Researchers have extensively studied the pathogenesis of HE and the treatments available. However, there are no studies examining how melatonin influences HE. In this review, we have described the precise molecular mechanism of HE and how melatonin protects against HE.

## 8. Conclusions and Future Prospects

HE is a severe neuropsychiatric hepatic disease that triggers various neuropathological alterations. Here, we summarized the functions of melatonin in HE neuropathology.

In HE, melatonin exhibited neuroprotective effects by increasing the enzyme activity involved in ammonia detoxification, by controlling liver enzymes, and by inhibiting ammonia’s entry into the brain by maintaining BBB integrity.

In the astrocyte, melatonin inhibits the conversion glutamate to glutamine by activating the ammonia detoxify enzymes and increasing the antioxidant enzymes’ level, ultimately decreasing the Ca^2+^ influx by melatonin, which leads to astrocyte swelling and brain edema. In the neuron, melatonin inhibits glutamine synthesis, proinflammatory cytokines, and inflammatory signaling pathways by activating free radical scavengers. This leads to decreased neuro-glial inflammation, insulin resistance, and the increased synaptic plasticity that is involving in cognitive function. Moreover, melatonin demonstrated potent hepatoprotective activity by regulating liver enzymes, reducing oxidative stress by increasing the antioxidant level, and decreasing inflammation in the HE liver. Here, we suggest the therapeutic potential of melatonin in the HE brain. Based on recent evidences, melatonin is involved in multiple neuroprotective responses in HE brains, including enhancing insulin sensitivity, modifying abnormal neurotransmitter and neuromodulator secretion, and reducing inflammatory responses and inhibiting BBB disruptions.

Although a limited number of studies have been attempted to investigate the effects of melatonin on HE, there are still few studies on the regulatory mechanisms of melatonin on neurotransmitters, cognition, and insulin regulation mechanisms in hepatic encephalopathy.

As part of this review, we described clinical and experimental studies conducted on melatonin and liver failure (Table 2), which increased the level of the antioxidant enzymes, reduced hyperammonemia, and hepato-neurotoxicity [1,191,192]. Numerous clinical studies demonstrated that liver diseases are associated with altered circulatory melatonin levels [25,193]. Mina Bahram et al. reported that administration of 6 mg melatonin had a refinement impact on non-alcoholic fatty liver disease (NAFLD) features such as imbalance anthropometric measurements, high blood pressure, abnormal liver enzymes, high sensitive C-reactive protein (hs-CRP), and abnormal leptin levels [194]. Moreover, melatonin is used as a therapeutic agent against obesity [195,196,197], obesity-induced leptin resistance [198,199], diabetes mellitus [200], hepatic steatosis [201], and myocardial injury [202].

Additionally, numerous studies have demonstrated the neuroprotective role of melatonin on liver diseases in vitro and in vivo models (Table 2). Various experimental results have demonstrated that melatonin exhibits antioxidative [203], anti-inflammatory [204], anti-hyperglycemic, and anti-apoptotic properties [205].

Given these clinical and experimental evidences, melatonin may be a new challenge for the treatment of HE neuropathology.

Further studies and clinical studies are needed to apply the appropriate melatonin therapy for brain damage following the progression of HE. Additionally, the monitoring serum melatonin level could be used as a predictive indicator of brain damage due to HE.

Hence, we suggest the possibility of using melatonin in combination with the existing drug treatment for HE and the melatonin alone treatment effect, and we expect to improve the quality of the life of patients with HE.

## Figures and Tables

**Table 1:** Effects of melatonin on hyperammonemia.

No.	Model	Type of Liver Injury	Methods	Experimental Findings	References
**1**	Hepato- and neurotoxicity induced by TAA/Adult Wistar rats	Melatonin (3 mg·kg^−1^·day^−1^) TAA (150 mg·kg^−1^ IP) Vitamin E (20 mg·kg^−1^) L-carnitine (100 mg·kg^−1^)	Liver (AST, ALT, LDH) Kidney (urea, BUN) Brain (ammonia, GSH, LPO)	Melatonin is a potent antioxidant that protects against TAA-induced hepato- and neurotoxicity compared to vitamins C and E	(Túnez et al., 2007) [51]
**2**	Hepato- and neurotoxicity induced by TAA/Adult Wistar rats	Melatonin (3 mg·kg^−1^ day^−1^) TAA (150 mg·kg^−1^ IP) DMSO (2 g·kg^−1^·day^−1^)	Liver (AST, ALT, LDH) Kidney (urea, BUN) Brain (ammonia, GSH, LPO)	Reduced hyperammonemia. Melatonin acts as an antioxidant and exerts neuro-/hepato-protective effects against TAA-induced hepato- and neurotoxicity	(Túnez et al., 2005) [49]
**3**	Adult male Wistar rats/ammonium acetate-induced brain damage	Ammonium acetate (100 mg/kg IP)—45 days Melatonin (5 mg/kg IP)/45 days	Biochemical analysis of oxidative stress and antioxidant markers in brain	Antioxidant property of melatonin protects against brain damage induced by hyperammonemia	(Lena & Subramanian, 2004) [52]
**4**	Adult male Wistar rats/ammonium acetate-induced brain damage	Ammonium acetate (100 mg/kg IP)—45 days Melatonin (5 mg/kg IP)/45 days	Biochemical analysis of non-enzymatic antioxidant markers in the brain	Antioxidant property of melatonin protects against brain damage induced by hyperammonemia	(Subramanian, 2003) [53]

**Table 2:** Effects of melatonin on HE with cognitive decline.

No.	Model	Type of Liver Injury	Methods	Clinical/Experimental Findings	References
**1**	CCl_4_-induced LF/Sprague–Dawley male rats	CCl_4_—0.2 mL twice per week via the intraperitoneal route for 5 months Melatonin-5 weeks after CCl_4_-induced LF (0.4 mg/kg/day)	Morris water maze	Melatonin treatment ⮚ Improved cognition and motor skills in LF rats.	(Haeger et al., 2019) [165]
**2**	BDL/Young male Sprague–Dawley rats	BDL—5 weeks BDL + melatonin (release melatonin pellet (5 mg) implanted in peritoneum)—4 weeks	Morris water maze Plasma liver enzymes (ALT, AST, direct bilirubin, Total bilirubin) BDNF (Plasma, PFC, HI)—ELISA Anti-ADMA—IHC	Melatonin effectively ⮚ Restored spatial acquisition and memory retention ⮚ Inhibited the level of ADMA in plasma, PFC, and dorsal HI ⮚ Upregulation of BDNF in the dorsal HI of BDL rats.	(Hsu et al., 2018) [32]
**3**	Clinical	Liver cirrhosis patients	**PHES:** DST, NCT-A and NCT-B, SDT, and LTT, TAVEC, CVLT) Serum IL-6, IL-8, blood ammonia, plasma cGMP, MRI scan, HI subfield volumes, and resting FC analysis	Episodic memory (learning and long-term memory) impairments Poor performance related to verbal learning and long-term memory (delayed recall) Lower performance related to episodic verbal memory was more apparent In volumetric analysis Decreased right fimbria volume Efferent axons of the pyramidal cells in the hippocampus emerge and converge to form the fimbria, a prominent band of white matter. Alterations in the output of hippocampus information due to alterations in the integrity of the fimbria. Reduced FC	(García-García et al., 2018) [8]
**4**	Clinical	Liver cirrhosis patients	Psychometric tests (MMSE, WAIS, NCT, BNT)	Alteration of consciousness, speech disturbances, asterixis, tremor, increased tendon reflexes, muscle tone, and ataxic gait. Patients with MHE: subclinical cognitive alterations	(Brodersen et al., 2014) [166]
**5**	Clinical	Liver cirrhosis patients	psychometric tests (DS, BD, NCT-A&B, and ICT.	Persistent and cumulative deficits in working memory, response inhibition, and learning	(Bajaj et al., 2010) [167]
**6**	BDL/Young male Sprague–Dawley rats	BDL—2 weeks BDL + Melatonin (500 μg/kg/d)—2 weeks BDL + Melatonin (1000 μg/kg/d)—2 weeks	Morris water maze Plasma liver enzymes (AST, ALT, Creatinine, ALP, ammonia, MDA, GSH/GSSH) Liver, brain cortex, and HI (MDA, GSH/GSSG)	Melatonin treatment ⮚ Improved spatial memory ⮚ Restored liver GSH/GSSG levels ⮚ Acts as antioxidant in the liver and brain (dose dependent)	(Huang et al., 2009) [33]
**7**	Clinical	Patients with liver cirrhosis and HE	NCT-A, DST, and SIP test	Impaired cognition Elevated level of melatonin in plasma and diurnal variation	(Velissaris et al., 2009) [25]

## Abbreviations

Ach, acetylcholine; ALF, acute liver failure; ALP, alkaline phosphatase; ALT, alanine aminotransferase; AMPA, α-Amino-3-hydroxy-5-methyl-4-isoxazolepropionic acid; AST, aspartate aminotransferase; ATP, adenosine triphosphate; AQP4, aquaporin 4; BBB, alood–brain barrier; BCRP, breast cancer resistance protein; BDNF, brain-derived growth factor; BD, block design; BDL, bile duct ligation; BNT, Boston naming test; BUN, blood urea nitrogen; cAMP, adenosine 3′,5′-cyclic monophosphate; CBF, cerebral blood flow; CCL2, C-C motif chemokine ligand 2; CCl4, carbon tetrachloride; Cd11b, Macrophage-1 antigen/Complement receptor; CFTR, cystic fibrosis transmembrane conductance regulator; cGMP, cyclic guanosine monophosphate; CNS, central nervous system; COX-2, Cycloxygenase-2; CSF, cerebrospinal fluid; CPS-1, carbamyl phosphate synthetase I; CVLT, California Verbal Learning Test; DAG, diacyl glycerol; DBI, Diazepam binding inhibitor; DMSO, Dimethylsulfoxide; DST, Digit Symbol Test; Eph/EphR, Ephrins/Ephrin-Receptor; ERK, extracellular signal-regulated kinase; FC, functional connectivity; GABA, gamma-aminobutyric acid; GAP-43, growth-associated protein-43; Gβγ, G protein–coupled receptors (βγ); GFAP, glial fibrillary acidic protein; GSH, glutathione; GSH/GSSG, reduced glutathione/oxidized glutathione ratio; GSK-3, glycogen synthase kinase-3; GWAS, genome-wide association studies; Hcit, homocitrulline; HCN, hyperpolarization-activated cyclic nucleotide-gated ion channel; HE, hepatic encephalopathy; H1HR, H1 histamine receptor; HHH, hyperornithinemia-hyperammonemia-homocitrullinuria syndrome; HI, hippocampus; HIMOT, Hydroxyindole-O-methyltransferase; HO-1, Hemoxygenase-1; ICT, inhibitory control test; IFN-γ, Interferon-γ; IHC, immunohistochemistry; IL, interleukin; InsR, insulin receptor; iNOS, nitric oxide synthase; IP3, Inositol-3-phosphate; IP, intraperitoneally; IRS-1, InsR substrate; LDH, lactate dehydrogenase; LF, liver fibrosis; LPO, lipid peroxidation; LTT, line tracing test; LTP, long-term potentiation; MAPK, mitogen-activated protein kinase; MDA, malondialdehyde; MHE, minimal hepatic encephalopathy; MMP-9, matrix metalloproteinase 9; MMSE, mini-mental state examination; MRI, magnetic resonance imaging; MT, melatonin receptors; MTNR1B, melatonin single nucleotide polymorphism receptor 2; NCT-A, number connecting test A; NADPH, nicotinamide adenine dinucleotide phosphate oxidase; Nrf2, Nuclear factor erythroid 2-related factor 2; NAS, N-acetyl serotonin; NCCa-ATP, Na-K-2Cl cotransporter; NF-κB, nuclear factor kappa B; NLRP3, nucleotide-binding domain (NOD)-like receptor protein 3; NMDA, N-methyl-D-aspartate; No, nitric oxide; Orn, ornithine; OS, oxidative stress; PD, Parkinson’s disease; PFC, Prefrontal cortex; PHES, Psychometric hepatic encephalopathy score; PKA, protein kinase A; PKG, protein kinase G; PLC, phospholipase; PP-2A, protein phosphatease-2A; PSD, postsynaptic density; PSE, porto-systemic encephalopathy; PTBR, peripheral benzodiazepine receptor; ROS, reactive oxygen species; RyR, ryanodine receptor; SA-β-Gal, senescence-associated β-d-galactosidase; SDT, serial dotting test; SIP, sickness impact profile; TAA, thioacetamide; TAVEC, Test de Aprendizaje Verbal España Complutense; TCA, tricarboxylic acid cycle; T2DM, Type 2 diabetic mellitus; TGFβ1, transforming growth factor β1; TJ, tight junction; TLR4, toll-like receptor 4; TNF, tumor necrosis factor; TrkBT, tyrosine receptor kinase B; WAIS, Wechsler adult intelligence scale.
